# Assessing the Variation in COVID-19 Severity Among the Different Nationalities Living in Qatar

**DOI:** 10.7759/cureus.58918

**Published:** 2024-04-24

**Authors:** Wafa Ibrahim, Razi Mahmood, Elmobashar Farag, Devendra Bansal, Mohamed Alfaki, Hamad E Al-Romaihi, Mohammed Al-Thani, Rohayu Binti Hami

**Affiliations:** 1 Epidemiology and Public Health, Advanced Medical and Dental Institute, Universiti Sains Malaysia, Penang, MYS; 2 Public Health, AFG College with the University of Aberdeen, Doha, QAT; 3 Epidemiology and Public Health, Ministry of Public Health, Doha, QAT; 4 Research, Sidra Medicine, Doha, QAT

**Keywords:** who region, infectious diseases, qatar, comorbidities, nationality, vaccines, covid-19, sars-cov-2

## Abstract

Background

Coronavirus disease (COVID-19) is a highly infectious disease caused by severe acute respiratory syndrome coronavirus 2 (SARS-CoV-2), and it has resulted in a global pandemic. The COVID-19 pandemic has resulted in numerous reports on clinical outcomes and risk factors associated with morbidity and mortality. However, the extent to which nationality influences the severity of COVID-19 is not fully understood. Therefore, this study aimed to explore disparities in COVID-19 severity among individuals of different nationalities in Qatar.

Methods

This is a retrospective study. Secondary data were obtained from the Ministry of Public Health in Qatar. Patients of different nationalities were categorized into different groups based on the WHO regional classification, and the severity of COVID-19 across these groups was analyzed.

Results

Data were obtained for 96,728 patients. This study found a statistically significant difference in disease severity among nationalities. The highest number of patients were from the Eastern Mediterranean group (42.3%), followed by Southeast Asia (39.4%). The severity of COVID-19 was highest among the Eastern Mediterranean groups (40%), followed by those from Southeast Asia (38.5%) and the Western Pacific (12.4%). There was a significant correlation between disease severity and vaccination status.

Conclusion

The findings of this study provide novel perspectives on the severity of COVID-19 among individuals of various nationalities. Moreover, it emphasizes the importance of healthcare interventions to address disparities in COVID-19 morbidity and mortality within these groups. The results of this study provide a useful foundation for developing approaches to prevent and manage pandemics more effectively and reduce the number of cases and fatalities during future health crises.

## Introduction

Coronavirus disease 2019 (COVID-19) is an infectious disease caused by severe acute respiratory syndrome coronavirus 2 (SARS-CoV-2), which was first given the name “2019 novel coronavirus." The disease was initially misdiagnosed as severe pneumonia and quickly became a global pandemic [1]. The first outbreak was reported in Wuhan, China, in December 2019, and it was linked to the Huanan seafood market [2]. The World Health Organization (WHO) was alerted to the new disease by Chinese health authorities on December 31, 2019, owing to its high transmission rate and potential to pose a global health threat [2,3]. As the pandemic progressed, reports on clinical outcomes and risk factors for ICU admission and mortality have emerged. The highly transmissible nature of COVID-19 has resulted in numerous studies examining the risk factors for severe outcomes, such as ICU admission and mortality. Although the role of nationality in COVID-19 severity is not yet fully understood, various studies have suggested that both genetic and socioeconomic factors may play a role. Additionally, age and comorbidities such as lung, heart, and metabolic diseases as well as obesity have been strongly associated with severe COVID-19 outcomes. Epigenetic alterations may also contribute to the onset of disease-related complications.

A study revealed that a high level of cytokines indicates a poor prognosis in COVID-19 patients. Additionally, postmortem examination of lung tissues from COVID-19 patients has revealed excessive infiltration of pro-inflammatory cells, involving macrophages and T-helper cells, which may contribute to the poor prognosis. Other studies suggest that a "cytokine storm" may increase the risk of mortality from COVID-19 [4]. The WHO reports that more than 700 million cases and six million deaths have been reported in 220 countries [5]. Qatar is one of the many countries affected by the COVID-19 pandemic. The population of Qatar is approximately 2.6 million, with foreigners comprising the majority. Qatari citizens account for only 12% (313,000) of the population, whereas the remaining 88% (2.3 million) have approximately 94 different nationalities. COVID-19 infections began in Qatar in February 2020, with the first cases identified among travelers returning to the country [6].

Qatar has granted emergency authorization for four vaccines: Pfizer-BioNTech, Moderna, AstraZeneca, and Pfizer-BioNTech for pediatric patients [4]. The vaccination process consists of two doses of the vaccine and a third booster dose. As of February 2022, 87.1% of the total population in Qatar had received at least one dose of the vaccine, while 77% of the population had been fully vaccinated [7]. According to studies conducted in Qatar between February and April 2020, there were 5,685 confirmed COVID-19 cases; 88.9% were male, and 8.7% were Qatari nationals. The highest number of infections occurred in patients from India (27.4%). Among these patients, 83.6% had no concomitant comorbidities, whereas only 3.0% had three or more comorbidities [6]. Qatar is a diverse multinational country with individuals from different ethnic backgrounds, each with unique behaviors, comorbidities, and immune system profiles. Further research is required to identify population-specific risk factors and evidence-based prevention and treatment strategies to reduce the incidence and mortality rate of COVID-19 in Qatar. While studies conducted in Europe and the USA have addressed the association between the severity of COVID-19 and potential risk factors such as ethnicity, no such studies have been conducted in the Middle East or Qatar. Consequently, the significance of nationality, which may affect disease transmission, prognosis, treatment, morbidity, and mortality rates in Qatar, remains unclear. Hence, additional research is necessary to understand and prevent COVID-19 in Qatar's multiethnic population.

This study was conducted to evaluate and disseminate information on the severity of COVID-19 among individuals of diverse nationalities residing in Qatar and identify potential risk factors. This study analyzed the impact of nationality on the severity and mortality rate of COVID-19 in Qatar. The findings of this study will contribute to a better understanding of the morbidity, mortality, and prognosis of COVID-19, aiding in assessing their implications in preventive and predictive medicine.

## Materials and methods

Research framework and ethical considerations: This is a retrospective cross-sectional study. It includes laboratory-confirmed COVID-19 cases reported by the Ministry of Public Health (MOPH), Qatar, between January 2021 and February 2022. The study included secondary data provided by the MOPH for patients who were discharged from the hospital or died. The vaccination status, nationality, age, gender, and comorbidities were recorded.

Ethical approval (ERC-826-3-2020) was obtained from the MOPH Ethical Review Board. The Human Research Ethics Committee USM (HREC), University of Sains Malaysia (JEPeM-USM), also reviewed and approved the study protocol (USM/JEPeM/PP/23020198).

Sample size: Stratified random sampling was used to ensure that all WHO regions were sufficiently represented for the appropriate statistical analyses. The researchers stratified the sample by the WHO region and used the Epitools software for a simple random sample within each group. Epitools is a type of sampling tool that researchers and epidemiologists utilize to estimate disease prevalence and randomly select individuals from a population without introducing selection bias [8].

Variables: Patient data were categorized into groups based on WHO regional classifications (Africa, America, Eastern Mediterranean, Europe, Southeast Asia, and the Western Pacific region) [9]. The severity of the disease was categorized into asymptomatic (no symptoms), symptomatic (symptoms but no ICU admission), and severe (symptoms + hospital/ICU admission or death).

Inclusion/exclusion criteria: The inclusion criteria for this study included all confirmed COVID-19 patients living in Qatar, including those who had been discharged from the hospital or had passed away. Nonresident patients were excluded from the study.

Statistical analysis: The data were analyzed using SPSS. Descriptive statistics were used to summarize the demographic characteristics of the patients. Severity in numbers and percentages was analyzed, and the chi-squared test was used to compare the severity of COVID-19 among patients from different regions living in Qatar. Multiple logistic regression analysis was performed to determine the association between vaccination and the severity of COVID-19 among different nationalities living in Qatar. The independent variables were age, gender, and chronic diseases, with disease severity as the dependent variable. The vaccination status was included as an independent variable.

## Results

**Figure 1:**
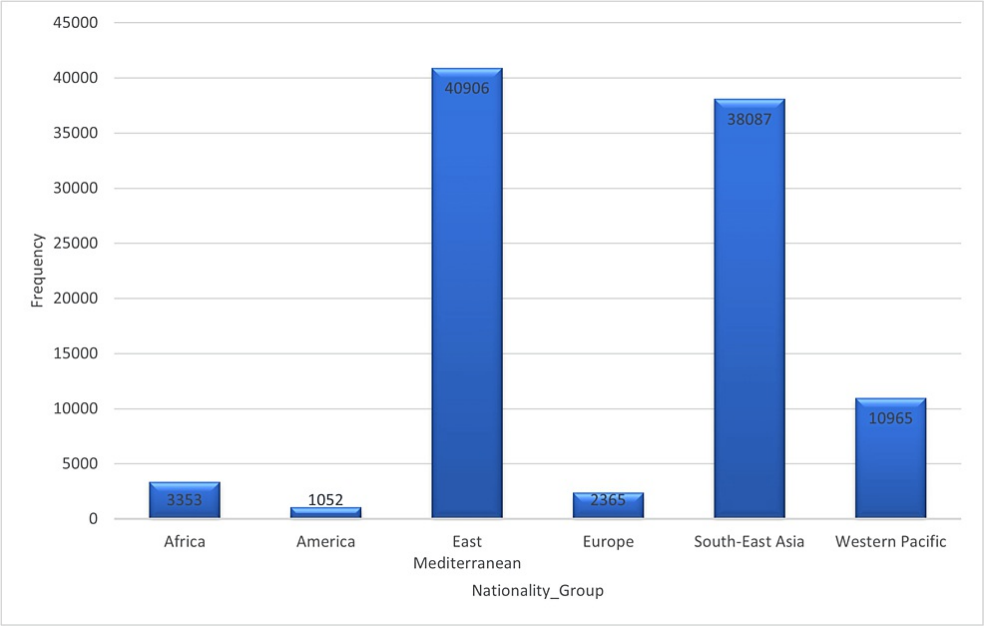
Number of COVID-19 cases among different nationalities

Demographics: Overall, data were obtained from 96,728 patients with confirmed COVID-19. A total of 94,739 data points were analyzed (1,989 individuals had missing data, representing 2.06%, and were excluded from the analysis). The results showed that the Eastern Mediterranean had the highest number of cases, 40,906 (42.3%), followed by Southeast Asia with 38,087 (39.4%), and the Western Pacific with 10,965 (11.3%) groups (see Figure 1). The countries with the most cases were India (24.8%), the Philippines (10.9%), and Nepal (6.6%). The 20-39 age group had the highest number of cases (39.7%), and the average age of the participants was 35.39 years (standard deviation: 15.5) (Table 1).

In this study, there were more males (65.2%) than females (34.8%). Most of the patients were asymptomatic (64.9%). The data also indicated that most patients (77.1%) were vaccinated, while 22.9% were unvaccinated. Among vaccinated individuals, the highest number of cases (44.9%) occurred after the second dose (Table 1).

**Table 1:** Study sample characteristics stratified by disease severity Note: Defined severity: asymptomatic (no symptoms), symptomatic (symptoms but no ICU admission), severe (symptoms + hospital/ICU admission); P value was calculated using the chi-square test. NS indicates no significance (p > 0.05). CVD: cardiovascular disease; SARS-CoV-2: Severe acute respiratory syndrome coronavirus 2; HTN: hypertension

		Severity				
		Asymptomatic	Symptomatic	Severe	Total	*P value
Nationality group	Africa	2,429	823	9	3,261	<0.001
	America	741	292	1	1,034	
	East Mediterranean	26,631	13,112	68	39,811	
	Europe	1,708	604	5	2,317	
	Southeast Asia	24,413	13,038	65	37,516	
	Western Pacific	6,684	4,095	21	10,800	
Gender	Male	40,887	20,736	115	61,738	NS
	Female	21,719	11,228	54	33,001	
Age group	0-19	12,399	4,943	30	17,372	<0.001
	20-39	25,142	12,321	63	37,526	
	40-59	22,369	13,091	69	35,529	
	≥ 60	2,696	1,609	7	4,312	
Vaccine status	Unvaccinated	15,662	5,991	38	21,691	<0.001
	First dose	390	260	3	653	
	Second dose	27,660	14,705	79	42,444	
	Booster	18,894	11,008	49	29,951	
Vaccine type	Null	15,662	5,991	38	21,691	<0.001
	SARS-CoV-2(AstraZeneca)	554	243	2	799	
	SARS-CoV-2 (Moderna)	14,647	8,524	45	23,216	
	SARS-CoV-2 (Pfizer)	31,621	17,164	83	48,868	
	SARS-CoV-2 (Pfizer) Pediatrics	122	42	1	165	
Comorbidities	Diabetes	5,118	4,095	14	9,227	<0.001
	HTN	5,739	4,392	22	10,153	<0.001
	CVD	1,109	808	4	1,921	<0.001
	Dyslipidemia	4,202	3,196	12	7,410	<0.001
	Asthma	2,329	1,751	8	4,088	<0.001
	Cancer	504	256	3	763	NS
	Kidney	504	396	3	903	<0.001
	Obesity	2,577	1,874	11	4,462	<0.001
Death	Death	21	10	42	75	<0.001

Table 1 shows that most patients received Pfizer (51.6%), followed by Moderna (24.4%) and AstraZeneca (0.8%).

The analysis revealed a significant association between severity and nationality (p < 0.001).

Hospital/ICU admission and death: A total of 426 patients were hospitalized, 169 of whom required ventilation, and 75 died. There were 31 deaths (41%) among patients aged 29-39 years (Appendix A).

Compared with individuals in other groups, the Western Pacific group reported the highest number of symptoms (7.9%). African patients experienced a slight increase in tracheostomies and ICU admissions (Table 2). The majority of fatalities occurred among Indian patients (23 deaths, 30.7%), followed by Qataris (nine deaths, 12%), Nepalese (eight deaths, 10.7%), and Filipinos (seven deaths, 9.3%) (Appendix B).

**Table 2:** Nationality group and disease outcomes (n=96,728) Note: There was a significant association between disease outcomes and different nationalities.

Nationality group	No. of cases		Asymptomatic		Symptomatic		Hospitalization		ICU/Tracheostomy		Death	
	N	%	N	%	N	%	N	%	N	%	N	%
Africa	3,353	3.5%	2,429	72.4%	823	24.5%	17	0.50%	9	0.3%	3	0.08%
America	1,052	1.1%	741	70.4%	292	27.7%	3	0.28%	1	0.1%	2	0.19%
East Mediterranean	40,906	42.3%	26,631	65.1%	13,112	32.1%	171	0.41%	68	0.2%	23	0.05%
Europe	2,365	2.4%	1,708	72.2%	604	25.5%	9	0.38%	5	0.2%	2	0.08%
Southeast Asia	38,087	39.4%	24,413	64%	13,038	34.2%	173	0.45%	65	0.2%	38	0.09%
Western Pacific	10,965	11.3%	6,684	61.9%	4,095	37.9%	53	0.48%	21	0.2%	7	0.06%
Total	96,728	100%	62,606	66.1%	31,964	33.7%	426	0.4%	169	0.2%	75	0.1%

Comorbidities: The most prevalent underlying medical conditions in COVID-19 patients were hypertension (10.7%), diabetes (9.7%), and dyslipidemia (7.8%). Patients from the Western Pacific region had the highest incidence of hypertension (19.4%), followed by those from Southeast Asia. Africans were the least vulnerable to hypertension. We also found that Southeast Asians had a higher prevalence of diabetes (12%) and cardiovascular disease (CVD) (2.5%) than other groups. The prevalence of obesity was highest among patients from the Eastern Mediterranean region (8.5%), while Americans had the highest incidence of dyslipidemia (9.1%). Patients from the Eastern Mediterranean region were also at a greater risk of asthma and chronic obstructive pulmonary disease (COPD) (6%) than those from other regions, with the American group showing a lower risk (3.8%). African patients had the lowest prevalence of comorbidities, such as hypertension, diabetes, CVD, dyslipidemia, asthma, and obesity, compared to other groups (Table 3). Our data showed that patients from the Eastern Mediterranean and Southeast Asian groups had a higher prevalence of severe COVID-19 (40% and 38.5%, respectively) than those from the Western Pacific region (12.4%) (Table 4).

**Table 3:** Common comorbidities among different nationalities

Comorbidities	Nationality groups												
	Africa		America		East Mediterranean		Europe		Southeast Asia		Western Pacific		P value
No. of cases	3,353	3.5%	1,052	1.1%	40,906	42.3%	2,365	2.4%	38,087	39.4%	10,965	11.3%	
HTN	89	2.7%	112	10.6%	3,348	8.2%	169	7.1%	4,499	11.8%	2,132	19.4%	<0.001
Diabetes	63	1.9%	65	6.2%	3,517	8.6%	96	4.1%	4,626	12%	1,044	9.5%	<0.001
CVD	5	0.1%	24	2.3%	750	1.8%	35	1.5%	934	2.5%	206	1.9%	<0.001
Dyslipidemia	31	0.9%	96	9.1%	3,383	8.3%	140	5.9%	2,977	7.8%	928	8.5%	<0.001
Asthma and COPD	38	1.1%	40	3.8%	2,470	6%	78	3.3%	1,156	3%	381	3.5%	<0.001
Cancer	11	0.3%	14	1.3%	379	0.9%	36	1.5%	190	0.5%	151	1.4%	<0.001
Kidney	8	0.2%	11	1%	422	1%	12	0.5%	331	0.9%	140	1.3%	<0.001
Obesity	27	0.8%	59	5.6%	3,483	8.5%	68	2.9%	701	1.8%	224	2%	<0.001

**Table 4:** Post-hoc analysis of severity by ethnicity Note: An adjusted residual > 1.96 indicates a statistically significant difference between the expected and observed counts.

		Severity			
Nationality groups		Asymptomatic	Symptomatic	Severe	Total
Africa	Count	2,429	823	9	3,261
	% within severity	3.9%	2.6%	5.3%	3.4%
	% of total	2.6%	0.9%	0.0%	3.4%
	Adjusted residual	10.3	-10.4	1.3	
America	Count	741	292	1	1,034
	% within severity	1.2%	0.9%	0.6%	1.1%
	% of total	0.8%	0.3%	0.0%	1.1%
	Adjusted tesidual	3.8	-3.8	-.6	
East Mediterranean	Count	26,631	13,112	68	39,811
	% within severity	42.5%	41.0%	40.2%	42.0%
	% of total	28.1%	13.8%	0.1%	42.0%
	Adjusted residual	4.5	-4.5	-.5	
Europe	Count	1,708	604	5	2,317
	% within severity	2.7%	1.9%	3.0%	2.4%
	% of total	1.8%	0.6%	0.0%	2.4%
	Adjusted residual	7.9	-7.9	.4	
South‒East Asia	Count	24,413	13,038	65	37,516
	% within severity	39.0%	40.8%	38.5%	39.6%
	% of total	25.8%	13.8%	0.1%	39.6%
	Adjusted residual	-5.3	5.3	-.3	
Western Pacific	Count	6,684	4,095	21	10,800
	% within severity	10.7%	12.8%	12.4%	11.4%
	% of total	7.1%	4.3%	0.0%	11.4%
	Adjusted residual	-9.8	9.8	4	
Total	Count	62,606	31,964	169	169
	% within severity	100.0%	100.0%	100%	100.0%
	% of total	66.1%	33.7%	0.2%	0.2%

Impact of Vaccination on COVID-19 Outcomes in Various Ethnic Groups in Qatar

The results of this study indicated that most COVID-19 patients were vaccinated (77.1%). Among vaccinated individuals, the highest number of cases (44.9%) was observed among those who received the second dose of the COVID-19 vaccine. Of the vaccinated patients, 51.6% received the Pfizer vaccine, followed by Moderna (24.4%), and AstraZeneca (0.8%) vaccines. There was a significant correlation between disease severity and vaccination status (Table 5). Disease severity was greater among individuals who received a single vaccine dose.

**Table 5:** Vaccination status among different ethnic groups *Statistically significant if the P value was <0.05, calculated using the chi-squared test.

		Severity				
Vaccination status	Ethnic group	Asymptomatic	Symptomatic	Severe	Total	*P value
0 (Not vaccinated)	Africa	984	252	5	1,241	< 0.001
	America	217	71	1	289	
	East Mediterranean	9,912	3,873	24	13,809	
	Europe	393	131	1	525	
	Southeast Asia	3,635	1,360	6	5,001	
	Western Pacific	521	304	1	826	
	Total	15,662	5,991	38	21,691	
First dose	Africa	27	10	0	37	< 0.001
	America	2	1	0	3	
	East Mediterranean	145	111	1	257	
	Europe	12	7	0	19	
	Southeast Asia	184	115	2	301	
	Western Pacific	20	16	0	36	
	Total	390	260	3	653	
Second dose	Africa	1,160	437	4	1,601	< 0.001
	America	271	103	0	374	
	East Mediterranean	12,677	6,754	34	19,465	
	Europe	666	231	3	900	
	Southeast Asia	10,718	5,957	31	16,706	
	Western Pacific	2,168	1,223	7	3,398	
	Total	27,660	14,705	79	42,444	
Third dose	Africa	258	124	0	382	< 0.001
	America	251	117	0	368	
	East Mediterranean	3,897	2,374	9	6,280	
	Europe	637	235	1	873	
	Southeast Asia	9,876	5,606	26	15,508	
	Western Pacific	3,975	2,552	13	6,540	
	Total	18,894	11,008	49	29,951	

Multinomial logistic regression analysis was performed with severity as the dependent variable and nationality, group, and sex as factors. The logistic regression model fit was significant (p < 0.05). The data show that nationality was a significant predictor (p < 0.05).

Parameter estimates: The reference category was symptomatic, and this parameter was set to zero because it was redundant. The addition of gender and ethnic group variables significantly improved model fit, as indicated by the likelihood ratio tests. The chi-square value for the gender variable was 14.292, with three degrees of freedom and a P value of 0.003, whereas the chi-square value for the ethnic group variable was 476.624, with 15 degrees of freedom and a P value less than 0.001 (Appendix C).

## Discussion

This study indicated a statistically significant difference in disease severity among different nationalities. The highest number of patients were from the Eastern Mediterranean group, followed by Southeast Asia. The severity of COVID-19 increased in Eastern Mediterranean individuals, followed by patients from Southeast Asia and the Western Pacific region. To investigate the factors contributing to the global spread of COVID-19, it is important to examine the potential influence of nationality on the disease. Therefore, we analyzed national surveillance reports and articles on COVID-19, focusing on race and ethnicity data and comparing them with surveillance data from Qatar. Many studies have linked poor outcomes of COVID-19 to different nationalities [10-14]. Our study included patients from various countries who received equal levels of healthcare within Qatar's health facilities. Our diverse sample comprised men and women of all ages, representing a broad range of exposure categories. In this study, the severity of COVID-19 varied from asymptomatic to severe.

We hypothesized that nationality and comorbidities would affect the severity of COVID-19. Apparent disparities in infection rates and clinical outcomes exist across different countries. However, there is still a lack of information regarding the effect of nationality on COVID-19 clinical outcomes. Therefore, we focused on the COVID-19 outcomes among residents of Qatar. Our findings revealed that individuals from the Eastern Mediterranean region had the highest percentage of COVID-19 cases, followed by those from Southeast Asia and the Western Pacific. These findings are significant because previous studies have shown that South Asian patients have the highest incidence of COVID-19 [14]. Certain groups, including South Asians, also present higher rates of comorbidities such as diabetes, hypertension, and cardiovascular diseases. These conditions are associated with severe disease and mortality in COVID-19 patients [3]. Our findings align with those of previous research, as we demonstrated that Southeast Asian patients had the highest rates of diabetes (12%) and CVD (2.5%), surpassing those of other groups. Additionally, we found that the majority of patients were males (65.2%), which is consistent with previous reports from China and Italy that also showed associations between risk factors such as sex, age, smoking status, cardiac comorbidities, and increased adverse effects [3]. However, mortality rates differ between Chinese and Italian populations, suggesting that race may impact outcomes [3]. Moreover, among African patients, we observed slight increases in severe symptoms, hospitalizations, and ICU admissions. This finding is consistent with a CDC report that showed that African Americans tend to have an incidence rate of approximately 2.1 times greater and a hospitalization rate of 4.7 times greater than those of the general population [10]. Other studies have demonstrated a significantly increased mortality rate for COVID-19 among Black and minority ethnic populations. In contrast, another study attributed the higher COVID-19-related mortality rate among African American, Black, and Hispanic ethnic groups to disparities in access to healthcare [11,12,14].

One study in Louisiana showed that Blacks accounted for 76.9% of hospitalized patients and 70.6% of deaths, while they constituted only 31% of the Ochsner Health population [13]. In contrast, our findings revealed that African individuals had the lowest comorbidity rate.

The findings of this study highlight the high risk of disease in specific groups, such as Southeast Asians, and the severity of disease associated with comorbidities such as hypertension, diabetes, and obesity. The data showed that patients from the Eastern Mediterranean and Southeast Asian groups had a higher prevalence of diabetes, CVD, asthma, and COPD than those from the other groups. These findings correlate with those of many studies that have found a relationship between comorbidities and disease severity progression in patients. These studies indicate that patients with comorbidities such as hypertension, diabetes, asthma, COPD, and chronic kidney disease were significantly affected in higher proportions, resulting in the requirement of ICU admission and ventilator aid in some cases. In many cases, this increases the risk of developing severe COVID-19 infections and increases the risk of severe clinical outcomes [15-17]. Moreover, several studies on the clinical efficacy and safety of COVID-19 vaccines have shown that the effectiveness of the coronavirus vaccine was 66% in preventing symptomatic disease, 88% for hospitalization, 90% for ICU admission, and 86% for preventing COVID-19-related deaths [18,19]. These findings are consistent with those of our study, which showed a significant correlation between disease severity and vaccination status, consistent with previous studies. Moreover, we observed that disease severity was greater among individuals who received only a single vaccine dose and waned after the second dose.

This study also revealed a significant association between nationality and deaths due to COVID-19. However, further research is needed to support this finding because of the limited sample size.

The results of this study may be utilized to compare disease outcomes among people of various nationalities residing in different environments and affected by various factors. By comparing the results of this study with data from other countries, it is possible to investigate a range of factors that may influence COVID-19 outcomes and contribute to these differences, this includes culture, genetics, age distribution, health policies, access to healthcare, and wealth. This comparison provides valuable insights into the unique characteristics of each population, which may influence variations in severity and mortality rates. Nonetheless, it is essential to acknowledge that the relationship between nationality and health outcomes is intricate and multifaceted.

Recommendations

We believe that further research should be conducted to evaluate the role of nationality in determining COVID-19 incidence and mortality and to identify screening methods, detection methods, and early response strategies.

Limitations of the study

Although this study revealed that certain nationalities may be at a higher risk of developing severe COVID-19 and other diseases, these findings cannot be generalized to all members of a particular group. Moreover, it is important to note that the countries in the WHO region may have different ethnicities. Further research is needed to evaluate the role of ethnicity in the outcomes of COVID-19, which will enable us to better understand the severity of the disease.

## Conclusions

This study provides new insights into the severity of COVID-19 among individuals of various nationalities. Furthermore, it emphasizes the importance of healthcare interventions to mitigate disparities in COVID-19 mortality within these groups. Understanding the impact of nationality on the severity of infectious diseases will enable the development of effective methods for detecting and responding to future pandemics, leading to improved national and international surveillance of infectious diseases.

## Appendices

**Table 6:** Appendix A, number of deaths per age

Age groups	Death	
0-19	Count	11
	% within death	14.7%
	% of total	14.7%
20-39	Count	31
	% within death	41.3%
	% of total	41.3%
40-59	Count	29
	% within death	38.7%
	% of total	38.7%
60->60	Count	4
	% within death	5.3%
	% of total	5.3%

**Table 7:** Appendix B, number of deaths per nationality in Qatar

Nationality	Number of cases	Number of deaths (75)	Mortality
American	487	2	2.7
Bangladesh	4,011	2	2.7
Filipino	10,562	7	9.3
Indian	23,954	23	30.7
Indonesian	579	2	2.7
Irish	62	1	1.3
Jordanian	2,326	1	1.3
Kenyan	1,220	1	1.3
Nepalese	6,407	8	10.7
Pakistan	3,984	4	5.3
Palestinian	1,021	2	2.7
Qatari	17,366	9	12
Saudi	446	1	1.3
Sri Lankan	3,061	3	4
Sudanese	2,566	1	1.3
Ugandan	336	1	1.3
Yemeni	1,700	2	2.7
Iranian	847	1	1.3
Bahrain	184	1	1.3
Egyptian	4,917	1	1.3
Ethiopian	541	1	1.3
Greek	54	1	1.3

**Table 8:** Appendix C, parameter estimates Note: a. The reference category is symptomatic. b. This parameter is set to zero as it is redundant.

Severity^a^		B	Std. error	Wald	df	Sig.	Exp (B)	95% confidence interval for exp (B)	
								Lower bound	Upper bound
Missing	Intercept	-3.138	0.083	1,438.774	1	0.000			
	Female	-0.155	0.050	9.484	1	0.002	0.857	0.776	0.945
	Male	0^b^			0				
	Africa	1.008	0.136	55.199	1	0.000	2.740	2.100	3.575
	America	0.420	0.256	2.701	1	0.100	1.522	0.922	2.511
	East Mediterranean	0.723	0.085	71.641	1	0.000	2.061	1.743	2.436
	Europe	0.675	0.170	15.840	1	0.000	1.965	1.409	2.740
	Southeast Asia	0.036	0.091	0.154	1	0.695	1.036	0.866	1.240
	Western Pacific	0^b^			0				
Asymptomatic	Intercept	0.509	0.021	576.451	1	0.000			
	Female	-0.039	0.015	6.594	1	0.010	0.962	0.934	0.991
	Male	0^b^			0				
	Africa	0.589	0.045	171.674	1	0.000	1.803	1.650	1.969
	America	0.440	0.072	37.451	1	0.000	1.553	1.349	1.788
	East Mediterranean	0.217	0.023	92.834	1	0.000	1.243	1.189	1.299
	Europe	0.549	0.051	114.206	1	0.000	1.731	1.565	1.914
	South‒East Asia	0.125	0.023	29.329	1	0.000	1.133	1.083	1.186
	Western Pacific	0^b^			0				
Severe	Intercept	-5.187	0.232	498.724	1	0.000			
	Female	-0.180	0.172	1.092	1	0.296	0.835	0.596	1.171
	Male	0^b^			0				
	Africa	0.743	0.400	3.441	1	0.064	2.102	0.959	4.608
	America	-0.410	1.025	0.160	1	0.689	0.664	0.089	4.952
	East Mediterranean	0.005	0.250	0.000	1	0.985	1.005	0.615	1.641
	Europe	0.474	0.500	0.902	1	0.342	1.607	0.604	4.278
	Southeast Asia	-0.083	0.257	0.105	1	0.746	0.920	0.556	1.522
	Western Pacific	0^b^			0				
